# The Impact of Female Genital Mutilation on Sexual Function: A Study Conducted in Rural Sudan

**DOI:** 10.7759/cureus.51343

**Published:** 2023-12-30

**Authors:** Mustafa Cengiz Dura, Salih Mahmoud Abaker Salih, Hilal Aktürk, Özgür Aslan

**Affiliations:** 1 Obstetrics and Gynaecology, Bakırköy Sadi Konuk Education and Research Hospital, Istanbul, TUR; 2 Obstetrics and Gynecology, Nyala Sudan Turkish Training and Research Hospital, Istanbul, TUR

**Keywords:** genital mutilation, sexual dysfunction, fgsis, fsfi, cutting

## Abstract

Backround: There are few studies comparing sexual function in women with female genital mutilation (FGM) in the literature, and most of these were evaluated with the Female Sexual Function Index (FSFI) questionnaire. Only one used the Female Genital Self-Image Scale (FGSIS) questionnaire.

Aim: This study aims to evaluate the effects of FGM on sexual function in Sudanese women who did or did not undergo FGM, using the FSFI and FGSIS questionnaires.

Methods: This descriptive study was conducted on Sudanese women from July 2020 to March 2021. Patients who attended to our hospital's gynecology outpatient clinic for health screening were included in this study. A total of 211 patients 113 with FGM and 98 without FGM were included in the study. The group with FGM was categorized according to the classification of the World Health Organization. The validated Arabic FSFI and FGSIS questionnaires were administered to groups with and without female genital mutilation and cutting (FGM/C).

Results: When the FGM types of the cases participating in the study were examined, patients with FGM were classified according to the FGM/C classification defined by the World Health Organization. They were classified as 20.4% (n=23) Type 1, 49.6% (n=56) Type 2, and 30.1% (n=34) Type 3. FSFI and FGSIS scores were significantly lower in the FGM/C group, especially in Type 3 with the highest tissue loss. The survey results statistically support the possibility of sexual dysfunction in FGM group.

Clinical implications: Female genital circumcision negatively affects sexual function. Therefore, clinicians should consider and sexual dysfunction in women with FGM attending primary care.

Strengths and limitations: The strengths of this study are its originality, as it is the first study in the literature to use validated FGSIS and FSFI questionnaires together to assess sexual function in groups with and without FGM and to evaluate correlation of questionnaire results. We undertook the study it using validated and reliable scales, trained clinical staff, local staff gynecologist, and multivariate analysis. Limitation of the study is the chosen age range. The reason for limiting the age to under 35 is that we wanted to evaluate the more sexually active age group in our study. We cannot comment on the correlation of FSFI and FGSIS in circumcised patients over 35 years of age.

Conclusion: Sexual function and sexual self-image of women with FGM/C were found to be significantly lower compared to women without FGM when compared with the validated FSFI and FGSIS questionnaires.

## Introduction

The World Health Organization (WHO) refers to the technique performed on the female vaginal area as "mutilation" [1]. Mutilation is defined as the infliction of a bodily damage with the intention of altering the form or function of any live body [2]. The WHO classified FGM into four types. Type 1, also known as clitoridectomy. Type 2 is defined as partial or complete removal of the labia minora and majora, as well as removal of the clitoris. Type 3, also known as infibulation, involves the excision of a portion or the entirety of the external genitalia, followed by the reapproximation of the remaining labia majora. Type 4 encompasses various forms of injury to the female genital organs. Female genital mutilation (FGM), a culturally rooted procedure prevalent in Africa and Asia, is typically carried out by traditional practitioners and affects over 200 million women worldwide [3,4].

There are approximately 15 million children under the age of 18 and 4.5 million children under the age of five in Sudan [5]. FGM was reported to be prevalent among Sudanese women aged 14 to 49 at a rate of 89% [6]. In Sudan, FGM is routinely performed, particularly among girls aged 6 to 12. Despite the fact that FGM was banned by the Sudanese government in 2020, it still continues to be commonly practiced. It is known that in many countries where FGM is prohibited; the practice is carried out clandestinely as the cultural beliefs behind the notion are not destroyed. Likewise, UNICEF's report involving 30 countries in Africa and the Middle East states that although there are legal regulations against FGM in 24 of these countries, it is still widely practiced. FGM is frequently performed by a midwife without anesthesia or the use of antibiotics [7].

There are few studies comparing sexuality of women with FGM in the literature. Most of these were evaluated with the Female Sexual Function Index (FSFI) questionnaire, and only one used the Female Genital Self-Image Scale (FGSIS) questionnaire to assess sexual function. In the region where we work and in other societies where FGM is practiced, the practice is accepted as a social norm. The FGSIS questionnaire is a woman's assessment of her sexual self-image. The reason we applied the FGSIS questionnaire together with the FSFI is to evaluate the correlation between the two. In case of correlation, it will be supported that although the society accepts this practice as the norm, it is not accepted as normal by the woman with FGM.

Rouzi et al., in their study, investigated whether there was a significant difference between FGSIS scores in women with and without FGM [8]. In the study by Mohamed et al., FSFI scores were compared in 215 women with and without FGM according to the WHO FGM classification [9]. While other limited studies in the literature examined sexual function in women with and without FGM, especially FSFI scoring, only FGSIS scoring was used in Rouzi et al.'s study. However, in this study, the subtypes of the WHO classification were confirmed by the declaration of patients.

FGM has the ability to leave a permanent mark on the lives and minds of individuals who have undergone it, and it is accountable for a variety of short- and long-term consequences, including sexual dysfunction. The determination of sexual satisfaction has posed challenges due to the delicate nature of the subject matter [10]. A survey conducted with individuals who have undergone FGM revealed that they reported experiencing orgasm [11]. Nevertheless, an examination of 1,836 Nigerian women who had undergone FGM revealed that the procedure, specifically Types 1 and 2, did not diminish sexual sensations or the frequency of sexual intercourse. However, it was correlated with a greater occurrence of abnormal vaginal discharge and pelvic pain [12]. An additional study demonstrated a notable impact on sexual desire, arousal, and orgasm among individuals who had undergone Type 3 infibulation, in comparison to those who had undergone a Type 1 procedure [13].

 Consequently, there is a substantial lack of knowledge regarding FGM and its influence on women's sexual function [14]. The primary objective of this study was to assess the impact of FGM on sexual functioning.

## Materials and methods

This descriptive study was conducted from July 2020 to March 2021. The Institutional Review Board and Community Ethics Committee approvals were obtained (approval number 03/903.07.03/689). Sudanese participants who attended to the obstetrics and gynecology outpatient clinic of our tertiary referral hospital for health screening were included in the study.

The women included in the study were grouped as those between the ages of 18 and 48 who were sexually active, without any systemic disease, with and without FGM. A total of 211 patients, 113 with FGM and 98 without FGM, were included in the study. Exclusion criteria from the study were as follows; women with systemic diseases and vulvar dermatosis, those who had previous pelvic surgery, and individuals who were not sexually active. Patients gave their written informed consent to participate in the study.

The women included in the study all filled out a questionnaire in relation to social and demographic characteristics (age, body mass index, age at first sexual intercourse, gravida, parity, marital status, education). Each participant received an oral and written description of the study from trained clinical staff. A native gynecologist, together with a native health professional female translator who enables us to communicate with patients in Sudan was available at the interview room to be used when clarification was required by women and to make sure all items were answered. In order to determine the FGM type according to the WHO classification, the pelvic examinations of the individuals who gave informed consent were performed together with a local gynecologist.

As defined by the WHO, four different types of FGM exist [15]. Type 1: clitoridectomy (removal of the clitoris hood with or without clitoris removal); Type 2: removal of the clitoris as well as partial or all of the labia minora; Type 3: clitoris, labia minora, and labia majora are removed, and the introitus may be stitched or made smaller with a very small outlet for menstrual and urine transit (another terminology for this is infibulation); Type 4: the female genitalia being injured through non-medical procedures. Examples include pricking, piercing, incising, scraping, and cauterization. The patients were examined and classified into four types according to WHO-FGM criteria.

Both groups of patients with and without FGM were provided with the FSFI and FGSIS questionnaires translated into Arabic and validated [16,17].

FSFI has been translated into more than 20 languages and has become the "gold standard" in assessing the female sexual function as well as acting as an indispensable instrument for clinical research on female sexual dysfunction [18]. This questionnaire consists of six dimensions (desire, lubrication, arousal, orgasm, satisfaction, and pain) and contains 19 questions. A high FSFI score is indicative of improved sexual function in the patient. FSFI scores classified into the following groups: normal sexual function (total FSFI score ≥ 26.55), mild risk for female sexual disorder (total FSFI score 18-26.55), moderate risk for female sexual disorder (total FSFI score 11-17), and severe risk for female sexual disorder (total FSFI score <10) [19].

In order to assess the female genital self-image in a more objective manner, the FGSIS questionnaire was developed in 2010 [20]. The questionnaire was translated into Arabic in 2014 and subsequently validated [17]. The total FGSIS score is calculated by adding the results of seven questions. Lastly, the overall score goes from 7 to 28 points and a higher total FGSIS score reflects a more favorable genital self-image.

Statistical analysis

NCSS 2020 Statistical Software (NCSS LLC, Kaysville, USA) was utilized for statistical analysis.The quantitative variables were represented by the mean, standard deviation, median, minimum, maximum, frequency, and percentage values. Data normality was assessed by Shapiro Wilks and Box Plot charts. Student's t-test was carried out for quantitative two-group evaluations with normal distribution. Oneway Anova test was used in the comparison of three groups and above and the Bonferroni test was done to determine the group causing difference. Whilst Mann Whitney-U test was conducted in relation to non-normally distributed variables in two groups, Kruskal Wallis test for three groups and above, and Dunn test detecting the difference-causing group were performed. Fisher's Freeman Halton test was used to compare qualitative data. The results were evaluated at the 95% confidence interval and with a significance level of p < 0.05.

## Results

During the eight months of study, 113 patients with FGM and 98 patients without FGM were included. On review of the FGM types of cases participating in the study, patients were categorized as 20.4% (n=23) Type 1, 49.6% (n=56) Type 2, and 30.1% (n=34) Type 3.

With respect to demographic characteristics of participants such as age, educational level, residence and parity, there were no statistically significant differences between the FGM group and the control group. The ages of participants ranged from 17 to 38; the mean is 27.02±5.35. BMI values of the cases varied between 19.3 and 37.8 kg/m^2^; mean BMI is 26.71±3.35 kg/m^2^. It is noteworthy that 42.5% of the women in the FGM group and 28.6% of the women who were not in the FGM group were only primary-school graduates. Age, BMI values, education, number of pregnancies and births of the cases did not demonstrate a statistically significant difference according to circumcision status.

There was a significant difference between the two groups in terms of age at first sexual intercourse (Table 1). The group that did not undergo FGM exhibited significantly higher scores on various sub-dimensions of the FSFI scale, including "Desire," "Arousal," "Lubrication," "Orgasm," and "Pain," as well as the overall total scores, in comparison to the group that had undergone FGM (Table 2).

**Table 1:** Comparison of descriptive characteristics based on circumcision status

		Uncircumcised (n=98)	Circumcised (n=113)	p
Age	Mean±SD	26.39±5.26	27.58±5.39	0.108
	Median (Min-Max)	27 (18-36)	28 (17-38)	
BMI (kg/m^2^)	Mean±SD	26.99±3.29	26.47±0.39	0.262
	Median (Min-Max)	26.9 (19.3-37.8)	26.8 (19.3-37.8)	
Educational status	Primary school	28 (28.6)	48 (42.5)	0.110
	Middle school	43 (43.9)	39 (34.5)	
	High school	27 (27.6)	26 (23)	
Gravida	Mean±SD	2.66±1.78	2.81±1.76	0.487
	Median (Min-Max)	2 (0-8)	3 (0-7)	
Parity	Mean±SD	1.34±1.02	1.37±0.98	0.775
	Median (Min-Max)	1 (0-4)	1 (0-4)	
Sexual intercourse age	Mean±SD	16.72±2.27	15.36±1.40	0.001
	Median (Min-Max)	16 (13-24)	15 (13-19)	

**Table 2:** Comparison of FSFI and FGSIS based on circumcision status FSFI: Female Sexual Function Index; FGSIS: Female Genital Self-Image Scale

		Uncircumcised (n=98)	Circumcised (n=113)	p
FSFI				
Desire	Mean±SD	4.66±0.80	4.12±0.91	0.001
	Median (Min-Max)	4.8 (3.6-6)	3.6 (1.2-6)	
Arousal	Mean±SD	4.92±0.74	4.36±1.00	0.001
	Median (Min-Maks)	5.4 (2.7-6)	3.9 (1.8-6)	
Lubrication	Mean±SD	4.87±0.85	3.24±0.79	0.001
	Median (Min-Max)	4.8 (2.7-6)	3.3 (1.5-5.1)	
Orgasm	Mean±SD	4.24±0.70	3.93±0.82	0.004
	Median (Min-Max)	4.2 (2.8-5.6)	4 (2-5.6)	
Satisfaction	Mean±SD	4.55±0.72	4.24±1.03	0.087
	Median (Min-Maks)	4.8 (2.8-6)	4.4 (1.2-5.6)	
Pain	Mean±SD	4.13±0.92	3.32±0.98	0.001
	Median (Min-Max)	4 (1.6-5.6)	3.6 (1.2-5.6)	
FSFI Total Score	Mean±SD	27.37±2.96	23.21±3.58	0.001
	Median (Min-Max)	28 (20.2-33)	23 (13.3-29.7)	
FGSIS Score	Mean±SD	24.76±2.48	22.16±3.40	0.001
	Median (Min-Max)	25 (19-28)	23 (16-28)	

The average FSFI score for the non-FGM group was 27.37±2.96, whereas the FGM group had an average FSFI score of 23.21±3.58. Participants in the group with FGM fall within the risk range of mild sexual disorders. Especially when the participants with Type 3 FGM and those without FGM are compared, a significant difference is observed between the FSFI scores (Cohen’s d effect size=2.92). The obtained outcome is situated within the range of large effect sizes, as classified by Cohen. No statistically significant difference was identified between the scores of participants in the "Satisfaction" sub-dimension of the FSFI based on circumcision status. Overall scores of the unmutilated participants from the FGSIS were revealed to be statistically remarkably higher than those with the mutilation.

A statistically significant difference was noted between the sub-dimension scores and total scores obtained from the FSFI according to FGM types. In pairwise tests determining the source of difference, participants with FGM Type 3 had significantly lower sub-dimension ratings than those with Types 1 and 2 (Table 3).

**Table 3:** Comparison of FSFI and FGSIS according to circumcision types FSFI: Female Sexual Function Index; FGSIS: Female Genital Self-Image Scale

		Circumcision Type			p
		Type 1 (n=23)	Type 2 (n=56)	Type 3 (n=34)	
FSFI					
Desire	Mean±SD	4.72±0.84	4.22±0.82	3.55±0.77	0.001
	Median (Min-Max)	4.8 (3.6-6)	3.6 (2.4-6)	3.6 (1.2-4.8)	
Arousal	Mean±SD	4.7±0.86	4.79±0.82	3.42±0.69	0.001
	Median (Min-Max)	5.4 (2.7-5.4)	5.4 (3.6-6)	3.6 (1.8-5.4)	
Lubrication	Mean±SD	3.69±0.79	3.35±0.68	2.76±0.75	0.001
	Median (Min-Max)	4.2 (2.4-5.1)	3.6 (2.4-5.1)	2.7 (1.5-4.2)	
Orgasm	Mean±SD	4.38±0.69	4.09±0.83	3.38±0.58	0.004
	Median (Min-Max)	4.4 (3.2-5.2)	4 (2.8-5.6)	3.6 (2-0.4)	
Satisfaction	Mean±SD	4.77±0.73	4.61±0.77	3.27±0.94	0.001
	Median (Min-Max)	5.2 (3.6-5.6)	4.8 (1.2-5.6)	3.6 (1.2-4.8)	
Pain	Mean±SD	3.57±0.86	3.46±0.84	2.93±1.16	0.014
	Median (Min-Max)	3.6 (1.6-4.4)	3.6 (1.6-5.6)	2.4 (1.2-5.6)	
FSFI Total Score	Mean ±SD	25.82±3.12	24.51±2.47	19.31±1.89	0.001
	Median (Min-Max)	26.8 (18.3-29.7)	24.3 (19.9-29.3)	19.9 (13.3-21.9)	
FGSIS Score	Mean±SD	24.13±3.31	23.34±2.68	18.88±1.95	0.001
	Median (Min-Max)	26 (16-28)	24 (17-28)	19 (16-23)	

FGSIS total scores varied significantly in relation to FGM type. The pairwise analyses to ascertain the difference demonstrated that participants with FGM Type 3 had significantly lower scores than those with FGM Types 1 and 2. The non-FGM and FGM groups FSFI and FGSIS scores were statistically correlated (Table 4).

**Table 4:** Relationship of FSFI and FGSIS in groups FSFI: Female Sexual Function Index; FGSIS: Female Genital Self-Image Scale

		Uncircumcised	Circumcised
FSFI- FGSIS	r	0.872	0.822
	p	0.001	0.001

## Discussion

Investigating the sexual issues of FGM-treated patients in conjunction with their of genital self-image should contribute more clearly to the evaluation of their sexual health. According to our knowledge, this is the first study to examine the correlation between sexual dysfunction and genital self-image in FGM patients. In our study, the correlation between the FSFI and FGSIS questionnaire scores was clear, and with increasing severity of mutilation, lower scores in both scales were demonstrated.

A prominent view of the existence of FGM is that it is a culturally developed social norm providing a sense of social interaction in which all parties are encouraged for the unity of men and women [21]. The underlying reason for this idea has been to interpret FGM as a coordinated practice used by families to prepare their daughters for future marriage. The fact that the age at first sexual age is lower in the participants with FGM may be due to the importance given to marriage. Successful reproduction of couples after marriage in natalist societies brings prestige to the family, and at the same time, marriage strengthens families' alliances and strengthens them within the community [22]. For these reasons, FGM is accepted as a norm by many communities, especially in African countries [22]. The result is that the tissue loss as a result of mutilation disrupts the genital anatomy and therefore the sexual physiology, but considering the FSFI correlated decrease in the FGSIS scores of the women who are mutilated, these women are not at peace with their genital self-images, although the society accepts it as the norm, it is an indication that they do not accept mutilation as the norm.

Although there are few studies in the literature evaluating the effect of FGM on sexual function, there is no study evaluating the effect on subgroups classified and typed by WHO with the combination of FSFI and FGSIS [23,24]. Contrary to our study, in Rouzi et al.'s study, no significant difference was reported when comparing FGM and non-FGM participants using the FGSIS questionnaire [8]. However, a questionnaire that could evaluate the sexual functions of validated patients with FSFI or similar was not used to compare the FGSIS scores obtained in this study. In addition, unlike our study, FGM types were reported by the participants themselves in this study.

Development of the FGSIS questionnaire allowed for a more objective evaluation of the status of female genital self-image in 2010 [25]. With the exception of the FSFI Desire domain, it was discovered in this study that female sexual function and female genital self-image were highly associated. Having a positive self-perception about one's genitalia is fundamental to having satisfying sexual experiences [26]. A substantial correlation has been reported between favorable genital self-image and healthy sexual function [27,28].

Evaluating sexual satisfaction has been complicated because of the sensitive nature of the subject [10]. According to a study of participants with FGM, they were able to reach orgasm . A study of 1,836 Nigerian women who had had FGM found that neither kind of the operation reduced sexual desire or sexual activity but was linked to an increase in abnormal vaginal discharge and pelvic pain [12]. However, these studies did not use validated questionnaires to assess sexual function. Another study revealed that those who underwent Type 3 infibulation were considerably altered in terms of sex drive, arousal, and orgasm compared to those who underwent a Type 1 FGM. The findings in all studies were not conclusive or completely convincing, but they did suggest a link between FGM and sexuality dysfunction. In our study, a significant decrease was observed in all parameters of FSFI and FGSIS scores, except for the Satisfaction sub-dimension, in the circumcised group compared to the control uncircumcised group. This result shows that sexual functions are significantly adversely affected in women with FGM, especially in Type 3. Despite the significant difference between the groups, the FGM group is classified as moderate sexual disorder according to the FSFI score. This is important as the scores are not dramatically low in any of the groups.

Another remarkable point in the study is that there was a statistically significant difference between the two groups in terms of age at first sexual intercourse. The lower rate in the circumcised group may be due to the more traditional approach of families to marriage. Because, like circumcision, it will protect the girl in terms of the concept of honor in marriage.

The sheer volume of females at risk and the severity of the resulting health problems is startling. While the incidence of FGM has decreased in some nations, the overall population is increasing, putting more girls and women at danger. The rising medicalization of the practice, which can reach as high as 70 % in a country like Egypt, is another cause for grave concern. FGM is a global health issue that requires more attention [25]. The society has forced the women accept FGM as the norm. The result of the study supports that FGM treated women do not accept this situation as the norm with their self-assessment.

Furthermore, there are several potential complications that may arise following FGM. These include dysmenorrhea dyspareunia chronic infections of the vagina and bladder, difficulties with urination, fibrosis, keloids, sebaceous cysts, vulvar abscesses, infertility, and challenges with pelvic examinations, sexual intercourse, and vaginal delivery.

This study does have some limitations. The data on FGM type and sexual function were collected through self-report questionnaires. This means that there is a risk of recall bias and social desirability bias. The authors acknowledge that FGM is a deeply embedded cultural practice and that the women in their study may have been reluctant to report negative experiences due to social pressures. This may have led to an underestimation of the true impact of FGM on sexual function and genital self-image.The study recruited participants from a convenience sample of women attending a gynecological clinic. This means that the results may not be generalizable to the wider population of women who have undergone FGM. The questionnaires used in the study were only available in English and Arabic. This may have excluded women who do not speak either of these languages. The sample size of the study was relatively small, which may have limited the power to detect statistically significant differences between the groups.

The strengths of this study are its originality, as it is the first study in the literature to use validated FGSIS and FSFI questionnaires together to assess sexual function in groups with and without FGM and to evaluate correlation of questionnaire results. We conducted it using a validated and reliable scale, trained clinical staff, local staff gynecologist, and multivariate analysis. The other strength of the study is the determined age range. The reason for limiting the age of 35 is that we wanted to evaluate the more sexually active age group in our study.

## Conclusions

Sexual function of circumcised women was found to be significantly lower compared to uncircumcised women using the validated FSFI and FGSIS questionnaires in the current study. The FGSIS and FSFI scores of the women included in the study were correlated. Although it is thought that FGM is accepted as the norm in the studied societies, it emerges that the women exposed to this procedure are uncomfortable with the self-image evaluations. More research is required to determine the impact of FGM on female sexual function and genital self-image.

## References

[1] Earp BD, Johnsdotter S (2021). Current critiques of the WHO policy on female genital mutilation. Int J Impot Res.

[2] World Health Organization (1996). Organization, W.H., Female genital mutilation: report of a WHO technical working group, Geneva, 17-19 July 1995. Female genital mutilation: report of a WHO technical working group, Geneva, 17-19 July 1995.

[3] UNICEF UNICEF (2013). Female Genital Mutilation/Cutting: a statistical overview and exploration of the dynamics of change. Female Genital Mutilation/Cutting: a statistical overview and exploration of the dynamics of change.

[4] Marea CX, Warren N, Glass N, Ahmed W, Pallitto CC (2023). Advancing the measurement of knowledge, attitudes and practices of health workers who care for women and girls who have undergone female genital mutilation/ cutting (FGM/C): a qualitative exploration of expert opinion. PLoS One.

[5] (2023). Female genital mutilation. https://www.who.int/news-room/fact-sheets/detail/female-genital-mutilation.

[6] Elduma AH (2018). Female genital mutilation in Sudan. Open Access Maced J Med Sci.

[7] Nour NM (2004). Female genital cutting: clinical and cultural guidelines. Obstet Gynecol Surv.

[8] Rouzi AA, Berg RC, Alamoudi R, Alzaban F, Sehlo M (2020). Female genital self-image in women with and without female genital mutilation/cutting in Jeddah, Saudi Arabia. Sex Med.

[9] Mohamed AH, Mohamud RY, Mohamud HA, Eraslan A, Gur M, Omar AA, Cimen S (2022). Somalian women with female genital mutilation had increased risk of female sexual dysfunction: a cross-sectional observational study. Sci Rep.

[10] Andersson SH, Rymer J, Joyce DW, Momoh C, Gayle CM (2012). Sexual quality of life in women who have undergone female genital mutilation: a case-control study. BJOG.

[11] Lightfoot-Klein H, Shaw E (1991). Special needs of ritually circumcised women patients. J Obstet Gynecol Neonatal Nurs.

[12] Okonofu FE, Larsen U, Oronsaye F, Snow RC, Slanger TE (2002). The association between female genital cutting and correlates of sexual and gynaecological morbidity in Edo State, Nigeria. BJOG.

[13] Thabet SM, Thabet AS (2003). Defective sexuality and female circumcision: the cause and the possible management. J Obstet Gynaecol Res.

[14] Nzinga AM, De Andrade Castanheira S, Hermann J, Feipel V, Kipula AJ, Bertuit J (2021). Consequences of female genital mutilation on women's sexual health-systematic review and meta-analysis. J Sex Med.

[15] Nour NM (2015). Female genital cutting: impact on women's health. Semin Reprod Med.

[16] Lahlou R, Rostom S, Hari A, Bensaoud N, Bouazzaoui L, Bahiri R, Hajjaj-Hassouni N (2013). AB0253 cross cultural adaptation and validation of the Arabic version of the self-assessment questionnaire of sexual function: the female sexual function index (FSFI). Ann Rheum Dis.

[17] Mohammed GF, Hassan H (2014). Validity and reliability of the Arabic version of the female genital self-image scale. J Sex Med.

[18] Sun X, Li C, Jin L, Fan Y, Wang D (2011). Development and validation of Chinese version of female sexual function index in a Chinese population-a pilot study. J Sex Med.

[19] Yıldız Ş, Cengiz H, Kaya C (2022). Evaluation of genital self-image and sexual dysfunction in women with vulvar lichen planus or lichen sclerosus. J Psychosom Obstet Gynaecol.

[20] Herbenick D, Reece M (2010). Development and validation of the female genital self-image scale. J Sex Med.

[21] Efferson C, Vogt S, Elhadi A, Ahmed Hel F, Fehr E (2015). BEHAVIOR. Female genital cutting is not a social coordination norm. Science.

[22] Tuttle L (2000). "Sacred and politic unions": natalism, families, and the state in old regime France, 1666-1789.

[23] Biglu MH, Farnam A, Abotalebi P, Biglu S, Ghavami M (2016). Effect of female genital mutilation/cutting on sexual functions. Sex Reprod Healthc.

[24] Berg RC, Denison E (2012). Does female genital mutilation/cutting (FGM/C) affect women’s sexual functioning? A systematic review of the sexual consequences of FGM/C. Sex Res Soc Policy.

[25] Al Awar S, Al-Jefout M, Osman N, Balayah Z, Al Kindi N, Ucenic T (2020). Prevalence, knowledge, attitude and practices of female genital mutilation and cutting (FGM/C) among United Arab Emirates population. BMC Womens Health.

[26] Amos N, McCabe M (2016). Positive perceptions of genital appearance and feeling sexually attractive: is it a matter of sexual esteem?. Arch Sex Behav.

[27] Berman L, Windecker MA (2008). The relationship between women’s genital self-image and female sexual function: a national survey. Curr Sex Health Rep.

[28] Berman L, Berman J, Miles M, Pollets D, Powell JA (2003). Genital self-image as a component of sexual health: relationship between genital self-image, female sexual function, and quality of life measures. J Sex Marital Ther.

